# Evaluation of an Object Detection Algorithm for Shrapnel and Development of a Triage Tool to Determine Injury Severity

**DOI:** 10.3390/jimaging8090252

**Published:** 2022-09-19

**Authors:** Eric J. Snider, Sofia I. Hernandez-Torres, Guy Avital, Emily N. Boice

**Affiliations:** 1 U.S. Army Institute of Surgical Research, JBSA Fort Sam Houston, San Antonio, TX 78234, USA; 2 Trauma & Combat Medicine Branch, Surgeon General’s Headquarters, Israel Defense Forces, Ramat-Gan 52620, Israel; 3 Division of Anesthesia, Intensive Care & Pain Management, Tel-Aviv Sourasky Medical Center, Affiliated with the Sackler Faculty of Medicine, Tel-Aviv University, Tel-Aviv 64239, Israel

**Keywords:** object detection, algorithm, automation, tissue phantom, shrapnel, foreign body, ultrasound, artificial intelligence, deep learning, triage

## Abstract

Emergency medicine in austere environments rely on ultrasound imaging as an essential diagnostic tool. Without extensive training, identifying abnormalities such as shrapnel embedded in tissue, is challenging. Medical professionals with appropriate expertise are limited in resource-constrained environments. Incorporating artificial intelligence models to aid the interpretation can reduce the skill gap, enabling identification of shrapnel, and its proximity to important anatomical features for improved medical treatment. Here, we apply a deep learning object detection framework, YOLOv3, for shrapnel detection in various sizes and locations with respect to a neurovascular bundle. Ultrasound images were collected in a tissue phantom containing shrapnel, vein, artery, and nerve features. The YOLOv3 framework, classifies the object types and identifies the location. In the testing dataset, the model was successful at identifying each object class, with a mean Intersection over Union and average precision of 0.73 and 0.94, respectively. Furthermore, a triage tool was developed to quantify shrapnel distance from neurovascular features that could notify the end user when a proximity threshold is surpassed, and, thus, may warrant evacuation or surgical intervention. Overall, object detection models such as this will be vital to compensate for lack of expertise in ultrasound interpretation, increasing its availability for emergency and military medicine.

## 1. Introduction

Emergency medicine commonly uses ultrasound (US) imaging for its portability and real-time capabilities to diagnose various injury types. A typical example is the detection of foreign bodies lodged in soft tissues following high-energy penetrating trauma such as gunshots or blast explosions [1,2]. The retention of foreign bodies, if undetected and unmonitored can lead to pain, infection, or vessel and nerve laceration. However, the majority of cases can be handled conservatively [3,4], and, in the context of large-scale warfare, remain in combat operation. It is not the presence of the shrapnel itself, as much as its threat to nearby neurovascular structures that determine the severity and urgency of the injury.

Identification of the presence of these foreign bodies, such as fragments of shrapnel, requires advanced training for the end user. Clear image acquisition typically requires knowledge of various injury types and anatomical landmarks, patient positioning, and hours of image analysis experience to properly identify shrapnel within the field of view. Image interpretation can therefore be complicated and localization of the shrapnel with respect to other vital anatomical landmarks is essential to effectively and correctly triaging patients.

Machine learning algorithms to aid in US image interpretation have been developed for a variety of applications, including detection of tumors or nodules [5,6] and classification of lung pathologies seen in COVID-19 [7]. Previous work from our lab has developed a classifier model to successfully detect the presence of shrapnel in phantom tissue, as well as swine tissue [8,9] and compare its performance to conventional models trained on ImageNet datasets [10]. While the conventional classification models and our model (ShrapML) produced high performance metrics, identification of the precise location of shrapnel within the individual US images was still difficult.

Objection detection models can be deployed to detect instances of visual objects in digital images (either static or video), classify that object, and produce a bounding box around the object. These deep learning detection algorithms typically fall into one of two classes: dual-stage (R-CNN, Faster R-CNN [11,12,13]) and single-stage (YOLO [14,15,16,17], SSD [18,19]). While dual-stage methods typically achieve higher detection accuracy, these models are slower and computationally intensive. The end goal for a shrapnel detection model is eventual integration into an ultrasound instrument for real-time detection. For this reason, a single-stage detector was identified (YOLOv3 [16]) and applied in this work.

Here, we developed an object detection model for the detection of shrapnel in phantom tissue as well as with typical neurovascular landmarks (vein, artery, and nerve fiber). To highlight the use case for object detection models in ultrasound imaging applications, we develop a triage metric for quantifying shrapnel proximity to neurovascular landmarks as identification of shrapnel and its proximity to neurovascular structures is a critical triage tool for the eventual integration into real-time ultrasound hardware.

## 2. Materials and Methods

### 2.1. Fabrication of a Tissue Phantom Mold

A previously developed tissue phantom design was based on dimensions of a human adult male thigh and modified for these experiments [9]. The mold was composed of three distinct compartments: bone, muscle layer, and a subcutaneous fat layer. The bone had a diameter of 26 mm, the muscle layer was 94 mm, and the fat layer was 120 mm. The 3D models were designed using computer aided design software (Autodesk Inventor, San Rafael, CA, USA) and fabricated using a fused deposition modeling 3D printer (Raise3D, Irvine, CA, USA) with polylactic acid (Raise3D, Irvine, CA, USA) filament. The muscle layer mold was two pieces which snapped together and contained a recess for the nylon bone to slot in the center. The mold was fitted with a lid and held closed with vise-grips and sealing tape (McMaster-Carr, Elmhurst, IL, USA). Similarly, the outer fat layer was designed with a recess for the bone and components that snap together.

### 2.2. Construction of Gelatin Tissue Phantom

The inner layer (muscle) was 10% (*w*/*v*) gelatin (Fisher Scientific, Fair Lawn, NJ, USA) dissolved in 2:1 solution of water and evaporated milk (*v*/*v*) (Costco, Seattle, WA, USA) and 0.25% (*w*/*v*) flour (H-E-B, San Antonio, TX, USA). The water and milk solution were heated to approximately 45 °C, to increase gelatin solubility. To mimic heterogenous properties seen in ultrasound views of muscle, agarose (Fisher Scientific, Fair Lawn, NJ, USA) components were also incorporated. Two subsets of 2% agarose solutions were produced, one with 0.80% flour and the second with 0.10% flour, to include both brighter and darker echogenic components to the muscle layer, respectively.

The tissue phantom was created sequentially so the inner layer (muscle) solidified first around the bone and was removed from the mold and placed within the outer layer (fat) mold. This allowed the outer layer to solidify around the inner layer and bone. The outer layer (fat) was the same gelatin solution with 0.10% (*w*/*v*) flour. Solidification occurred at 4 °C for approximately 1 h for each layer. Afterwards, the completed tissue phantom was removed from the mold and used for shrapnel insertion and ultrasound imaging.

### 2.3. Incorporation of Vascular Vessels and Nerves

A modified phantom design was also used for this study which incorporated vein, artery, and nerve fiber features within the tissue phantom. This was performed after the base phantom model was constructed. First, the base phantom was divided into quarters and depending on bone positioning, the individual quarters were labeled as a right or left leg. One of the quarters that had vein, artery, and nerve simulating a right leg, contained shallow vessels near the interface between the fat and muscle layers, while the second quarter contained slightly deeper vessels fully in the muscle layer. The same methodology applied to the quarters that simulated the left leg. Then, the artery channels were created using a circular 8 mm biopsy punch (McMaster-Carr, Elmhurst, IL, USA) and oval-shaped biopsy punch with a minor axis of 6.5 mm for the vein. The oval-shaped biopsy punch was manually augmented from an 8 mm circular biopsy punch (McMaster-Carr, Elmhurst, IL, USA) by mechanical compression. Next, the nerve cavity was created lateral to the artery with the same biopsy punch as the vein and was filled with 15% gelatin made in 2:1 (*v*/*v*) water to evaporated milk ratio solution with 0.5% (*w*/*v*) flour. The ultrasound probe was aligned transversely for views of both right leg and left leg orientation. When the nerves were solidified, the phantom was considered ready for use with shrapnel insertion and ultrasound imaging.

### 2.4. Ultrasound Imaging with Shrapnel

All imaging was performed under water with the HF50 probe (Fujifilm, Bothell, WA, USA) from the Sonosite Edge ultrasound system (Fujifilm, Bothell, WA, USA). For both phantom designs, baseline images were obtained as frames from 15 s ultrasound video clips. For shrapnel fragments, we used a 2.5 mm diameter brass rod (McMaster Carr, Elmhurst, IL, USA) cut into fragments of varying length ranging from 2 mm to 10 mm. Shrapnel was inserted using surgical forceps at varying depths. For the modified, neurovascular phantom shrapnel was placed at similar depths but care was taken to avoid the shrapnel hitting the vein, artery, or nerve. Ultrasound imaging for shrapnel positive phantoms followed the same process as was performed for baseline imaging. Only out of plane images were used for the modified phantom so that neurovascular features could be identified. Both in plane and out of plane images were used for the base phantoms.

### 2.5. Image Processing and Bounding Boxes

Frames of all video clips were extracted using an implementation of FFmpeg with a Ruby script, yielding 90 individual frames per video. Duplicate frames were removed, and all images were processed with MATLAB’s image processing toolbox (MathWorks, Natick, MA, USA) in which a function was written to crop images to remove ultrasound settings from view and then resize them to 512 × 512 × 3. MATLAB also was used for the manual addition of bounding boxes to all images for all the objects. Individual rectangular boxes were drawn enclosing the smallest area around the shrapnel, vein, artery, or nerve (*n* = 6734 images base phantom; *n* = 10,777 images modified neurovascular phantom).

### 2.6. ShrapOD Architecture

**Figure 1:**
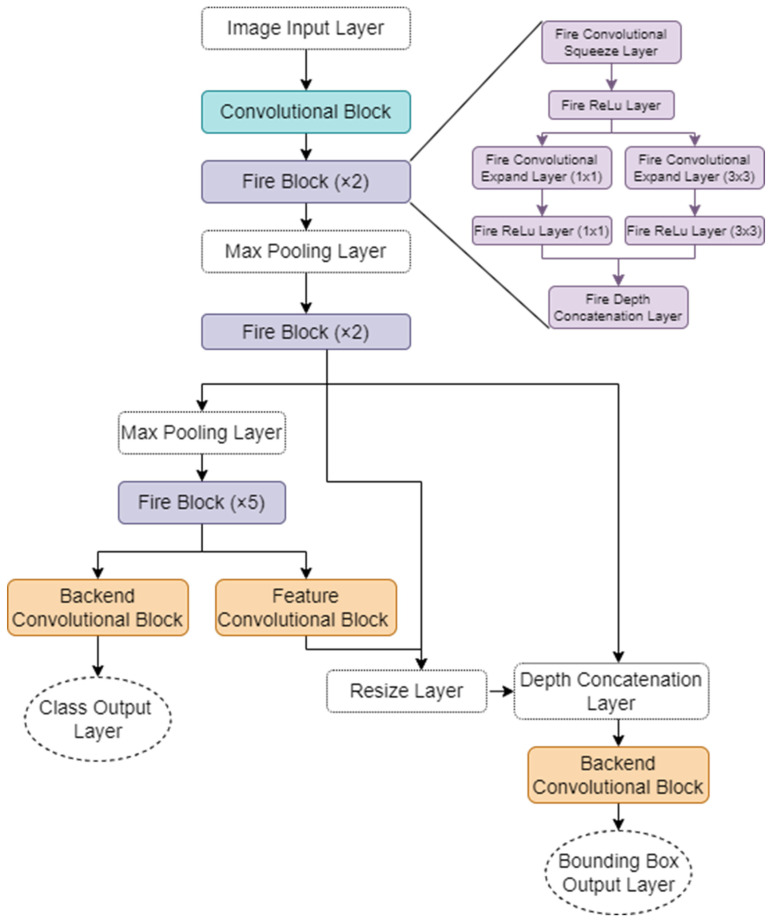
Basic overview of ShrapOD model network architecture. Diagram for the object detection algorithm using SqueezeNet [20] as the classification backbone, with added YOLOv3 outputs [16] to generate bounding boxes and class predictions is shown. In the diagram, individual layers are shown as well as “blocks” that consist of multiple layers. The convolutional block (teal) has a convolution layer, ReLU activation layer, and a max pooling layer. The Fire blocks (purple—that repeat two or five times as indicated) begin with a convolution layer with ReLU activation and then split into parallel chains with varying convolution filter sizes (1 × 1 and 3 × 3) with ReLU activation. As depicted in the first occurrence, then the parallel chains come back together using a depth concatenation layer. The feature convolutional block and both backend convolutional blocks are identical in layer content, beginning with a convolutional layer, followed by a batch normalization and ending with a ReLU activator. Both output layers, for class and bounding box are also convolutional layers.

The object detection model, ShrapOD, used a SqueezeNet neural network backbone [20] with modifications to include YOLOv3 object detection heads [16], as shown in Figure 1. This network architecture was built based on MATLAB-provided object detection code [21]. The feature extraction network in SqueezeNet was modified to use an image input layer of (512 × 512 × 3) followed by a convolution block containing a convolution layer with ReLU activation and max pooling layer. This is followed by 4 Fire blocks prior to the network splitting to integrate the YOLOv3 object detection heads. Fire modules, per the SqueezeNet architecture [20], comprised a single convolution squeeze layer (1 × 1, ReLU activator) followed by expanding layers consisting of a mixture of (1 × 1) and (3 × 3) convolutional layers in parallel to increase the depth and width for higher detection accuracy. These parallel layers are concatenated prior to the next layer in the network architecture to reduce the number of model parameters. Five additional Fire blocks are used on the YOLOv3 class output layer pathway, followed by a convolutional layer with batch normalization and ReLU activation (left pathway, Figure 1).

An additional output layer was used for bounding box predictions in which the network was fused after the Fire block 9 concatenation step with Fire block 8 with an additional convolutional block for feature resizing. The model contained a final concatenation layer and convolution block to align the predicted bounding box coordinates to the output image (right pathway, Figure 1). The YOLOv3 network also used optimized anchor boxes to help the network predict boxes more accurately [21].

### 2.7. ShrapOD Training Overview

**Figure 2:**
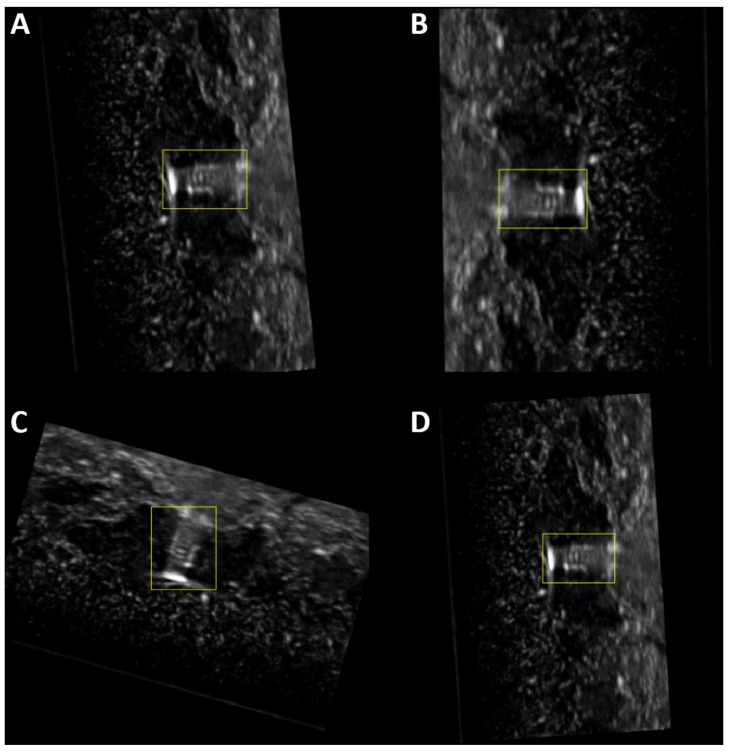
Augmented Shrapnel Images and Corresponding Bounding Boxes. (**A**–**D**) Single representative images having undergone random image augmentation four times. Image augmentation included X and Y flips or reflection, rotation, and zoom or scaling. All image augmentation performed to a single image was applied to the corresponding bounding box(es) as well as shown.

Model training was performed using MATLAB R2022b with the deep-learning and machine-learning toolboxes for the base phantom and then repeated for the modified, neurovascular phantom. For the base phantom use case, only images containing shrapnel were used. For the neurovascular phantom, images were taken from datasets with and without shrapnel. Images were cropped to remove ultrasound file information, sized to 512 × 512 × 3 and then datasets were split into 75% training, 10% validation and 15% testing quantities. Augmentation of the training datasets included random X/Y axis reflection, +/− 20% scaling, and +/− 360° rotation (Figure 2). These augmentation steps were written into a function that also applied it to the bounding box data. Validation and testing set images were not augmented. Training was performed using a stochastic gradient descent with momentum (SGDM) solver, 23 anchors, 125 epochs, L2 regularization of 0.0005, with a penalty threshold of less than 0.5 Intersection over Union (IoU), validation frequency of 79 iterations, and an image batch size of 16 images. The learning rate started at 0.001 and, after a warmup period of 1000 iterations, began a scheduled slowdown by $learning\;rate\times {\left(\frac{iteration}{warmup\;period}\right)}^{4}$. Training parameters were adapted from MATLAB object detection example code [21]. All training was performed using the CPU on an HP workstation (Hewlett-Packard, Palo Alto, CA, USA) running Windows 10 Pro (Microsoft, Redmond, WA, USA) and an Intel Xeon W-2123 (3.6 GHz, 4 core, San Clara, CA, USA) processor with 64 GB RAM.

### 2.8. Evaluating ShrapOD Performance

After ShrapOD model training, blind test (15%) images were used to measure model performance. For the ShrapOD model trained on the original phantom image sets (shrapnel only object class), 1010 images were used for testing while 1617 images were used in the multi-object trained model from the neurovascular phantom image sets. Predictions were compared to ground truth images to generate precision-recall curves using the evaluateDetectionPrecision function in MATLAB. The area under the precision-recall curve was found for determining average precision (AP) [22,23]. For intersection over union (IoU), a bboxoverlay function (MATLAB) was used for all test images. While calculating IoU scores, true positive (TP) counts were identified as having a prediction and ground truth with an IoU score greater than or equal to 0.50. False positive (FP) and false negative (FN) counts were based on this same IoU criteria of 0.50 for when no prediction exceeded this threshold and a ground truth was present or there was a prediction without a ground truth, respectively. Additionally, false positives were counted when multiple predictions for a single ground truth were detected. Precision, recall, and F1 scores were then calculated with this IoU gating of 0.50. Mean IoU (mIoU) scores were calculated across each object class, and, for the multi-object model, mean AP (mAP) and an average mIoU across all object classes was determined.

### 2.9. Triage Metric Measurement

**Figure 3:**
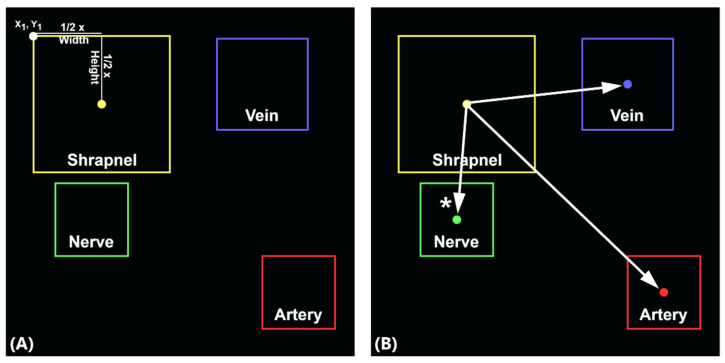
Diagram of triage metric calculation. (**A**) Measurements for the triage metric were based on object detection predictions for images with shrapnel, vein, artery, and nerve predictions. Prediction coordinates were recorded as (X_1_, Y_1_, Width, Height) where X_1_ and Y_1_ refer to the top left corner of the prediction window as shown. Midpoints of each prediction window, indicated by colored dots for each bounding box, were determined by adding ½ the width to the X_1_ coordinate and adding ½ the height to the Y_1_ coordinate. (**B**) Distance formula was used to calculate the distance (in pixels) between the shrapnel prediction and vein, artery, and nerve features. The minimum distance indicates which neurovascular feature is closest to shrapnel, which is the value selected as the triage metric; for this example, the shrapnel-nerve distance would be the minimal distance (as indicated by *, in pixel units).

For a medical use-case for object detection prediction, a triage metric score was developed that tracked the smallest distance between shrapnel and vein, artery, or nerve predictions (Figure 3). After test predictions were acquired, images were filtered to select only predictions where shrapnel, vein, artery, and nerve predictions were present. For images with multiple predictions for a category, only the highest confidence prediction window was used. Midpoints for each point were calculated by adding ½ the width and height to the X_1_ and Y_1_ coordinate, respectively (Figure 3A). Next, distances between shrapnel and vein, artery, and nerve were calculated using Equation (1).

(1) $$distance=\sqrt{{\left(x_{2}-x_{1}\;\right)}^{2}+{\left(y_{2}-y_{1}\;\right)}^{2}}$$

In Equation (1), $x_{2}$ and $y_{2}$ refer to midpoint coordinates for the shrapnel prediction window, and $x_{1}$ and $y_{1}$ refer to midpoint coordinates for the vein, artery, or nerve prediction windows (Figure 3B). Three distances were calculated, in pixel units, and the minimum distance was selected as the measurement for the triage metric for each ultrasound image. Results were compiled across filtered test images for further analysis.

## 3. Results

### 3.1. ShrapOD Performance for Tracking Shrapnel in Ultrasound Images

**Figure 4:**
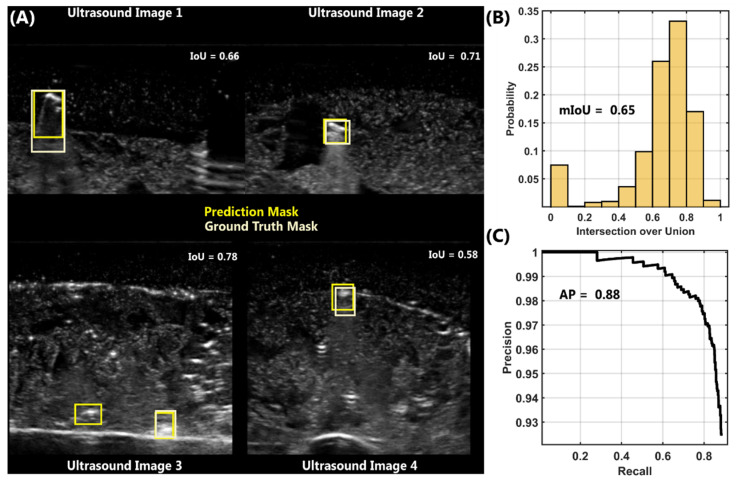
Results for Object Detection in Shrapnel-only Phantom Model. (**A**) Prediction (yellow) and ground truth (light yellow) shrapnel box overlays for four representative ultrasound images in the test data set. IoU scores for each image are shown on each image. (**B**) Probability distribution of IoU scores for test shrapnel data sets. Data is distributed into 10 bins with a mean IoU score of 0.65. (**C**) Precision-recall curve for shrapnel identification by the object detection model. Average precision was 0.88 for this model. Note, the *y*-axis is zoomed in to 0.93 to 1.0 to better highlight the changes in precision.

Using YOLOv3, training was performed on 75% of the total image set for 125 epochs. After training was completed, ShrapOD was evaluated on test image holdouts to determine its performance. By overlaying predictions and ground truth masks on US images, a qualitative inspection of images showed predictions were similar in location and size to ground truth images (Figure 4A). Predictions were not identical, with the box size sometimes being too large (Ultrasound Image 1, Figure 4A) or finding additional shrapnel objects (Ultrasound Image 3, Figure 4A). Overall results for these predictions were determined by intersection over union (IoU) scores to quantify the overlap in the predictions and ground truths. IoU scores ranged from 0 to 1 but was most prevalent in the 0.6 to 0.8 score range, with a mean IoU (mIOU) score of 0.65 (Figure 4B). A 0 IoU score indicated an image where no prediction was made (Percent False Negative (FN) = 6.8%) or a prediction was made that had no overlap with the ground truth (False Positive (FP) = 5.9% images). In addition, a precision-recall curve was generated for the model, and precision remained high for all confidence thresholds, resulting in an overall average precision (AP) of 0.88 (Figure 4C).

### 3.2. Results of Multi-Class ShrapOD Model for Shrapnel, Vein, Artery, and Nerve Identification

**Figure 5:**
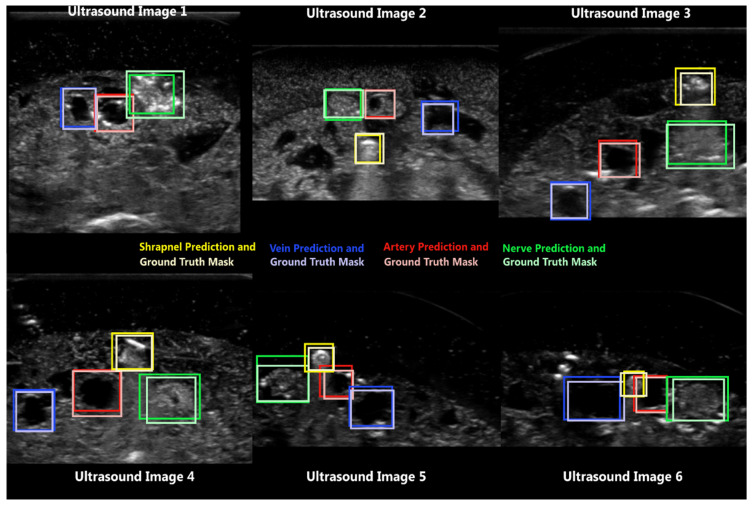
Representative predictions by ShrapOD for modified neurovascular phantoms with and without shrapnel present. Ultrasound Image 1 does not contain shrapnel while the rest have shrapnel present at different distances to the neurovascular features. Shrapnel (yellow), vein (blue), artery (red), and nerve (green) predictions are shown with ground truth overlays shown as fainter, lighter boxes in the same color scheme.

Next, the same network architecture was trained for use with multi-class object detection for shrapnel, vein, artery, and nerve using approximately 8000 images with ground truth masks identified. After 125 epochs, the model was evaluated with approximately 1600 test images held-out from the training. Ultrasound images with prediction and ground truth overlays for the multi-class object detection model were first evaluated as a qualitative “sanity” check on model performance (Figure 5). Only approximately 60% of images had shrapnel present, and the model successfully recognized this in most predictions (Ultrasound Image 1, Figure 5). Shrapnel varied in orientation and proximity to the other neurovascular features to add additional training complexity, sometimes easily identifiable away from other features (Ultrasound Image 2–3, Figure 5) and other times being much closer in proximity (Ultrasound Image 4–6, Figure 5).

**Figure 6:**
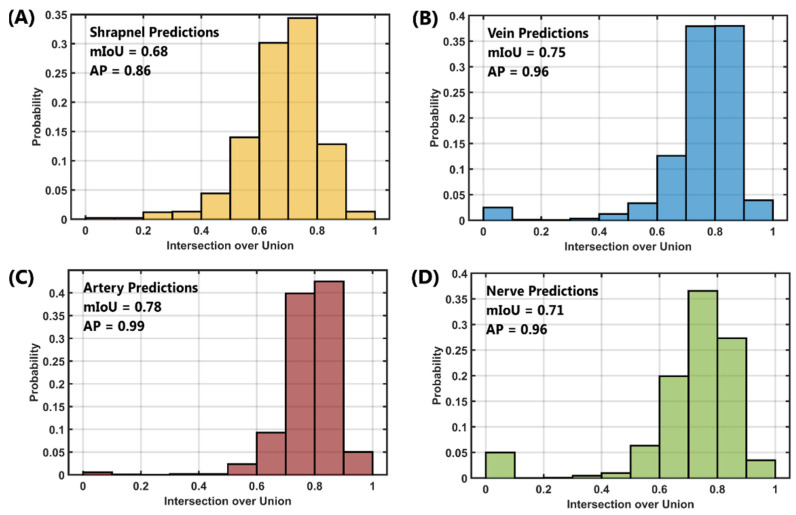
Intersection over Union Scores for ShrapOD with the Neurovascular Phantom. Probability distribution plots for IoU across each test data set are shown for (**A**) shrapnel, (**B**) vein, (**C**) artery, and (**D**) nerve. Mean IoU and average precision scores are shown for each prediction category.

Intersection over union and average precision were calculated for each feature category separately. IoU scores were first calculated across all test images (Figure 6) where a prediction or ground truth mask were present. Shrapnel predictions had a mIoU of 0.68 (FP = 7%, FN = 0%) with an AP of 0.86 (Figure 6A). Next, for the vein object-class, a mIOU of 0.75 (FP = 2%, FN = 2%) was achieved with an AP of 0.96 (Figure 6B). Artery features were more pronounced than other features in the model and as a result had a strong mIoU score of 0.78 (%FP = 1%, %FN = 0%) with an average precision of 0.99 (Figure 6C). Lastly, for the nerve, mIoU was 0.71 (FP = 2%, FN = 5%) with an AP of 0.96 (Figure 6D). This performance was stronger than anticipated as nerve bundles were harder to manually identify in images compared to the vein and artery. Across all classes, the average mIoU was 0.73 and mean AP was 0.94. A summary of the % TP, %FP, %FN, mIoU, precision, recall, F1 score, and average precision are captured in Table 1 for shrapnel, vein, artery and nerve and then an average of all object types.

### 3.3. Triage Metric Evaluation

**Figure 7:**
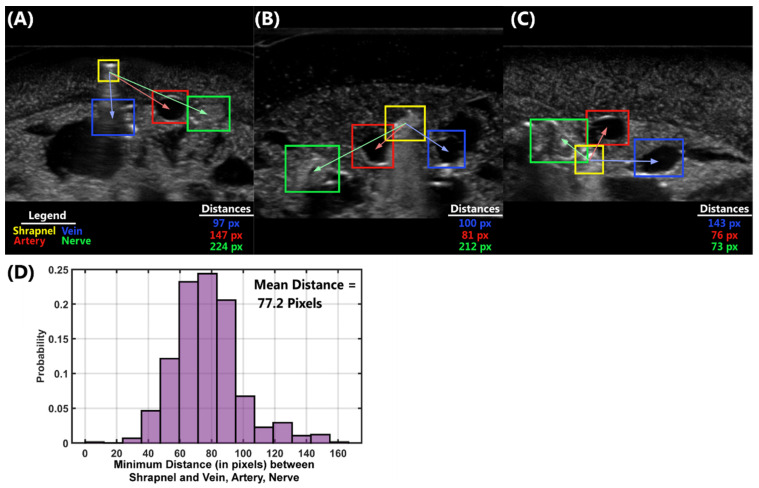
Results for the triage metric determined from the multi-class ShrapOD. (**A**–**C**) Distance measurements from center point of shrapnel to each neurovascular feature for three different ultrasound images of varying distances to shrapnel. Only prediction masks are shown (shrapnel = yellow, vein = blue, artery = red, and nerve = green) and distances, in pixels, from shrapnel to the vein (blue), artery (red), and nerve (green) are shown. (**D**) Probability distribution of minimal distances for each test image containing predictions for all four features. The mean minimal distance across all test images is shown.

Lastly, we demonstrate a use case for the multi-class ShrapOD model in ultrasound images by defining a triage metric for assisting during emergency or military medicine. This metric was defined as the minimal distance between the center of the shrapnel prediction and the three neurovascular features—vein, artery, or nerve. Representative images and approximate distance overlays are shown in Figure 7A–C. Distances—in pixel units—were calculated between shrapnel and each neurovascular feature. For simplicity, this initial use case for the triage metric, only test images where all four prediction categories existed were used and, for images where multiple single class predictions were made, only the higher confidence prediction was used. Mean distances were calculated for all test images that met these criteria (*n* = 758 images) and a probability distribution plot was generated (Figure 7D). The mean minimal distance was 77.2 pixels, and the vein, artery, and nerve frequency of being closest to shrapnel was 17%, 48%, and 29%, respectively (Table 2). The maximum distance to each feature was consistent at ~300 pixels while the closest minimal distance was for the nerve feature at an approximate 11-pixel distance to the shrapnel (Table 2). For highlighting how this measurement could be used in a medical triage decision, a gating window was arbitrarily selected based on the average ground truth window size for the artery—which was ~75 pixels. If all predictions where the triage metric distance was smaller than this threshold were identified as needing urgent medical attention, for this test image set, 46% of images would be flagged. However, the size of the window can be increased to 1.25× artery size to result in 80% of images or reduced to 0.75× to result in only 12% of images meeting the triage criteria (Table 2). Based on medical expertise and with adding real-life units, this metric can be a means of identifying the specific cases in which the shrapnel is lodged too close to key anatomical features, thus warranting further investigations and potentially surgical intervention. A summary of the mean distances from vein, artery, and nerve to shrapnel as well as other statistics are shown in Table 2.

## 4. Discussion

Ultrasound imaging is frequently used during emergency and battlefield medicine, thanks to its high portability and immediate nature. Interpreting images and using that information to prioritize care in resource limited situations remains a challenge that AI can potentially mitigate. Simple image classification algorithms have been developed to provide binary decisions from an ultrasound image, but that does not provide enough granularity in some use cases where the proximity of the tumor or shrapnel to other anatomical structures may be critical for decision making in the next steps for medical care. Here, we have highlighted how an object detection framework for shrapnel and multi-class tracking in ultrasound imaging can be used for assisting in emergency or military medicine triage, potentially avoiding unnecessary evacuation of the majority of shrapnel casualties from the battlefield.

First, we show that the YOLO object detection framework was able to be successfully trained for tracking only shrapnel in a base phantom design that we have previously used for developing image classification algorithms [8,10]. The tissue phantom allows for collection of shrapnel images at different locations, sizes, orientations across multiple phantoms created specifically for this purpose, introducing subject variability in the data set [9]. This is an ideal starting spot for deep learning model development and is possible for use with real-time deployment of the object detection model. Overall, the ShrapOD model was successful for detecting the approximate shrapnel location with a true positive rate of 87% and a mIOU of 0.65. With larger, more diverse image sets, these scores can likely be improved. The performance error is split evenly between false positives (6%) and false negatives (7%), suggesting it was missing shrapnel in the phantom as frequently as it was misidentifying complexities in the tissue phantom as shrapnel. However, more training and improvement on the object detection model was not performed in this work as this was an initial proof-of-concept use case.

Instead, we added physiological features to the tissue phantom by introducing a vein, artery, and nerve fiber bundle to mimic key neurovascular complexities. The original phantom mimics fat, muscle, and bone tissue in a thigh, so these neurovascular components are logical additions to more closely mimic anatomical composition of human tissue. Furthermore, this anatomical complexity provided a challenging, multi-class scenario for the object detection model. Training on these image sets resulted in stronger model performance for all four classes, with a mAP of 0.94. mIoU for the vein, artery, and nerve surpassed 0.7 while shrapnel was similar to the single class model at 0.68. Shrapnel is intentionally more irregularly present in the phantom due to different imaging angles compared to the other features, which likely contribute to the lower mIoU score. Similarly, like in real tissue, the nerve fiber bundle is more similar than the artery and vein in terms of echogenic properties to the tissue bulk, and, as anticipated has a lower mIoU score (0.71) when compared to the vein (0.75) and artery (0.78). While ShrapOD was successful at tracking multiple classes, this likely will require additional transfer learning to successfully predict in animal or human image cases. Supplementing phantom image sets with animal images was previously successful for training shrapnel classification models. However, the neurovascular features in the phantom are more spaced out than often found in real tissue, due to limitations in phantom design which may impact transfer-learning for this model.

For medical applications, especially in emergency situations, simply identifying where shrapnel is in tissue still leaves a subjective decision for a medical provider to make—remove the shrapnel or not. As this decision often relies on proximity to key vital structures—mostly neurovascular structures—the triage metric introduced in this work provides a practical use case for object detection. This was defined as the minimal distance from the center shrapnel prediction to the center of neurovascular features. This operation is simple enough that it could be paired with real time object detection model deployment to allow for proximities to key features to be automatically tracked. Further, while not evaluated in the current study, by calibrating this distance to a reference length, it can allow for real-life units of measurement instead of arbitrary pixel values. This metric can be further automated by setting an approximate distance for gating the distances against which alerts the end user. However, this would require additional clinical knowledge to determine a “critical threshold” of shrapnel distance from key structures that indicates an increased risk and may warrant surgical intervention.

One limitation for this triage use case is it solely relies on accurate tracking of up to four objects by the model—incorrect prediction of any prediction will result in incorrect triage distances, so fully automating will require a more robust image training set and rules for identifying poor prediction by the ShrapOD model. Another limitation is the triage metric is based on 2D ultrasound images, so objects may be out of plane from key neurovascular features not currently being tracked without 3D ultrasound information. In previous studies, 3D ultrasound volumes have been used for deep learning algorithms to more accurately track objects [24,25]. However, this metric is not meant to replace additional medical evaluation, but highlight at risk subjects, so the 2D tracking is likely sufficient. Lastly, all predictions are based on midpoints currently, which for larger vein diameters or more irregularly shaped shrapnel may misrepresent feature proximity. This can be improved by making measurements from each corner of the bounding boxes, or an image segmentation architectures may prove to be more applicable for improving on this metric [26].

Next steps for this work will take four paths. First, customization to the hardware side to allow object detection and triage measurement to be performed in real-time. Second, the ShrapOD model must be transfer learned for use with ex vivo or live animal tissue for further evaluation and for inclusion of pulsatile flow in the artery as it may impact object detection. Further, additional tissue and object tracking, such as proximity to joints, will be critical as this work is optimized for use in humans and translated into swine. Third, ultrasound image interpretation is only one half of the challenge when using ultrasound in military medicine; the other half of the challenge is proper image acquisition by non-expert users. This work will investigate further AI and robotic platforms to further automate the ultrasound process to make this technology more suitable for frontline application where resources and personnel are limited. Lastly, object detection algorithms can be valuable for other ultrasound applications so work will be conducted to develop AI models for other emergency medicine applications, such as eFAST (extended focused assessment with sonography in trauma) exams [27,28] for identifying pneumothorax [29], hemothorax, and abdominal hemorrhage.

## 5. Conclusions

In conclusion, artificial intelligence has the potential to revolutionize ultrasound imaging for emergency and military medicine by lowering the expertise level required for proper image acquisition and interpretation. Here, we demonstrated a use case for conventional object detection deep learning algorithms for identifying shrapnel as well as vital vein, artery, and nerve features in a tissue phantom. This enables a practical application of automating the measurement of shrapnel proximity to vital features in tissue that may be used for triaging whether evacuation or surgical intervention is needed. Further expansion of this technology can be used for other high priority emergency, military medical applications to make ultrasound a more practical triage, diagnostic tool even when skilled radiographic personnel are not present.

## Figures and Tables

**Table 1:** Summary statistics for the multi-object ShrapOD. Percent true positive (TP), false positive (FP), false negative (FN), mIoU, precision, recall, F1 scores, and average precision (AP) for all object types (shrapnel, vein, artery, nerve) and then averaged across all object types. TP, FP, and FN were calculated using a minimum IoU score 0.5 for accurate predictions.

	Shrapnel	Vein	Artery	Nerve	Average
Percent True Positive	93%	96%	99%	94%	95%
Percent False Positive	7%	2%	1%	2%	3%
Percent False Negative	0%	2%	0%	5%	2%
mIoU	0.681	0.753	0.784	0.709	0.732
Precision	0.928	0.978	0.992	0.983	0.970
Recall	1.000	0.979	0.999	0.952	0.982
F1	0.963	0.979	0.995	0.967	0.976
AP	0.858	0.955	0.991	0.957	0.940

**Table 2:** Summary table of statistics for triage metric. Distances to each of three features (in pixels), and percentage of images that would be flagged for five different gating windows, based on an average artery size of ~75 pixels in the images.

	Vein		Artery	Nerve	
Mean Distance to Shrapnel	131.1		91.3	120.5	
Frequency Closest to Shrapnel	17%		48%	29%	
Maximum Distance to Shrapnel	361.4		324.9	297.8	
Minimum Distance to Shrapnel	34.0		25.4	11.0	
	*Gating for Triage Metric, relative to ~75 pixel* *Average Artery Diameter*				
	0.50×	0.75×	1.00×	1.25×	1.50×
Triage Threshold	1%	12%	46%	80%	89%

## Data Availability

The datasets generated during and/or analyzed during the current study are available from the corresponding author upon reasonable request.
